# Identification of multi-drug resistant *Pseudomonas aeruginosa *clinical isolates that are highly disruptive to the intestinal epithelial barrier

**DOI:** 10.1186/1476-0711-5-14

**Published:** 2006-06-08

**Authors:** Olga Zaborina, Jonathan E Kohler, Yingmin Wang, Cindy Bethel, Olga Shevchenko, Licheng Wu, Jerrold R Turner, John C Alverdy

**Affiliations:** 1 Department of Surgery, University of Chicago, Chicago, USA; 2 Department of Pathology, University of Chicago, Chicago, USA; 3 Clinical Microbiology Laboratories, University of Chicago, Chicago, USA

## Abstract

**Background:**

Multi-drug resistant *Pseudomonas aeruginosa *nosocomial infections are increasingly recognized worldwide. In this study, we focused on the virulence of multi-drug resistant clinical strains *P. aeruginosa *against the intestinal epithelial barrier, since *P. aeruginosa *can cause lethal sepsis from within the intestinal tract of critically ill and immuno-compromised patients via mechanisms involving disruption of epithelial barrier function.

**Methods:**

We screened consecutively isolated multi-drug resistant *P. aeruginosa *clinical strains for their ability to disrupt the integrity of human cultured intestinal epithelial cells (Caco-2) and correlated these finding to related virulence phenotypes such as adhesiveness, motility, biofilm formation, and cytotoxicity.

**Results:**

Results demonstrated that the majority of the multi-drug resistant *P. aeruginosa *clinical strains were attenuated in their ability to disrupt the barrier function of cultured intestinal epithelial cells. Three distinct genotypes were found that displayed an extreme epithelial barrier-disrupting phenotype. These strains were characterized and found to harbor the *exoU *gene and to display high swimming motility and adhesiveness.

**Conclusion:**

These data suggest that detailed phenotypic analysis of the behavior of multi-drug resistant *P. aeruginosa *against the intestinal epithelium has the potential to identify strains most likely to place patients at risk for lethal gut-derived sepsis. Surveillance of colonizing strains of *P. aeruginosa *in critically ill patients beyond antibiotic sensitivity is warranted.

## Background

The human opportunistic pathogen, *Pseudomonas aeruginosa*, is a major cause of infectious-related mortality among the critically ill patients, and carriers the highest case fatality rate of all gram-negative infections [1]. Although the lungs have been traditionally considered to be a major site of *P. aeruginosa *infection among critically ill patients, a significant number of these infections arise as a result of direct contamination of the airways by the gastrointestinal flora or by hematogenous dissemination from the intestine to the lung parenchyma [2,3]. Yet even in the absence of established extraintestinal infection and bacteremia, the presence of highly virulent strains of *P. aeruginosa *within the intestinal tract alone can be a major source of systemic sepsis and death among immuno-compromised patients [4,5]. Extensive studies on the endemicity and prevalence of *P. aeruginosa *in the critically ill patients have identified the intestinal tract to be the single most important reservoir for this pathogen in cases of severe life-threatening sepsis [6,7]. Work from our laboratory has demonstrated that a major mechanism of the lethal effect of intestinal *P. aeruginosa *lies in its ability to adhere to and disrupt the intestinal epithelial barrier [8].

Within as little as 3 days in an intensive care unit, the feces of more than 50% of patients will culture positive for *P. aeruginosa *with up to 30% of these strains being antibiotic resistant [6]. In such patients, intestinal colonization by *P. aeruginosa *alone has been associated with a 3-fold increase in mortality in critically ill patients [4]. In fact the importance of intestinal *P. aeruginosa *as a cause of mortality in critically ill patients was recently demonstrated by a randomized prospective study in which selective antibiotic decontamination of the digestive tract (SDD) in critically ill patients with oral non-absorbable antibiotics decreased mortality associated with a decrease in fecal *P. aeruginosa *[9].

How multi-drug resistant (MDR) *P. aeruginosa *clinical isolates behave against the human intestinal epithelium is unknown. Therefore the purpose of this study was to determine the ability of MDR *P. aeruginosa *to disrupt epithelial integrity of Caco-2 monolayers and to correlate these findings to other relevant virulence features of *P. aeruginosa *including adhesiveness, motility, ability to form biofilm, and the presence of specific type III secretion related genes *exoU *and *exoS*.

## Methods

### Bacterial isolates

Under IRB protocol #11646B, University of Chicago, 35 strains of *P. aeruginosa *were consecutively obtained from the clinical microbiology laboratory from those selectively screened for gentamicin (Gm) resistance. We initially screened consecutive *P. aeruginosa *isolates that were resistant to Gm since Gm resistance has been shown to be the most common feature of MDR *P. aeruginosa *[10]. Among the 35 strains, three (# 3, 5, and 32) lost their resistance to Gm and one (#24) was re-identified not to be *P. aeruginosa *on subsequent culture. Therefore 31 clinical strains were available for phenotype and genotype analysis. Most isolates identified as *P. aeruginosa *were oxidase positive, hydrolyzed acetamide and arginine, oxidized glucose, and grew on cetrimide agar. Remaining isolates were identified by the Vitek 2 system (bioMérieux, Inc. Durham, NC). Additionally, isolates were verified by amplification of 16S DNA using primers forward 5'-GGACGGGTGAGTAATGCCTA-3' and reverse 5'-CGTAAGGGCCATGATGACTT-3', and genome DNAs of clinical isolates as templates. Susceptibility testing was performed by testing on the Vitek 2 or by disk diffusion. Susceptibility results were interpreted using Clinical Laboratory Standards Institute (CLSI) guidelines. Single colonies were picked up from Columbia SB agarized plates (Beckton Dickinson, Cockeysville, MD), grown in *Pseudomonas *broth containing Gm, 50 μg.ml^-1 ^and kept at -80°C as frozen stocks containing 8% glycerol. The isolates were routinely subcultured from frozen stocks on *Pseudomonas *isolation agar (PIA) containing Gm, 50 μg.ml^-1^. *P. aeruginosa *strains PAOI, ATCC 27853, PA103, and the environmental isolates PA190 and PA180 [11-13] were used as reference strains.

### DNA fingerprint analysis

The clonality of *P. aeruginosa *isolates was determined using the random amplified polymorphic DNA (RAPD) PCR fingerprinting, described previously [14-16]. Primers 208 (5'-ACGGCCGACC-3') and 272 (5'-AGCGGGCCAA-3') were synthesized and used in PCR amplifications. Intact bacteria were used as a source of template chromosomal DNA. The following protocol was used: 45 cycles of 1 min at 94°C, 1 min at 45°C and 1 min at 72°C. After the last cycle, samples were maintained at 72°C for 10 min. The resulting amplified DNA fragments were separated on agarose gels (0.8%, w/v) containing ethidium bromide (0.5 μg.ml^-1^) and visualized using UV radiation. Fingerprints were considered distinct if they differed by at least three bands.

### Human epithelial cells and transepithelial resistance (TER) assay

The Caco-2bbe (brush border-expressing) cell line was used in bacterial-cell culture experiments. Caco-2 cells were grown in 0.3 cm^2 ^transwells (Costar) in HEPES buffered (15 mM) DMEM media containing 10% FBS for 20 days, and electrophysiological measurements were done using agar bridges and Ag-AgCl-calomel electrodes and a voltage clamp (University of Iowa Bioengineering, Iowa City, IA) as previously described [17]. Fixed currents of 50 μA were passed across Caco-2 monolayers, and transepithelial resistance (TER) was calculated using Ohm's law. Fluid resistance was subtracted from all values. In order to assess the disrupting ability of *P. aeruginosa *strains against Caco-2 monolayers, overnight culture was added to the apical well (volume = 200 μl) to achieve a final bacterial concentration of ~10^7 ^CFU/ml. Media from the apical wells was then quantitatively cultured on PIA plates to determine the final bacterial count. Caco-2 monolayers were co-incubated with bacteria for up to 8 hours at 37°C, 5% CO_2_, and TER was measured each hour. All experiments were performed in triplicate.

### Swimming motility

Swimming assay was performed as previously described by Rashid and Kornberg [18]. Briefly, swim plates prepared by using of 1% tryptone, 0.5% NaCl and 0.3% (wt/vol) agarose, were inoculated with bacteria using a sterile toothpick. The plates were then wrapped to prevent dehydration and incubated at 37°C, overnight. The ability to swim was assessed by the radius of colony. All experiments were performed in triplicate.

### Twitching motility

Twitching motility was determined by the method of Rashid and Kornberg [18]. Fresh prepared and briefly dried twitch plates (Tryptic soy broth solidified with 1% (wt/vol) Difco granulated agar) were stab inoculated with a sharp toothpick into the bottom of the Petri dish. After incubation at 37°C for 24 h, the halo zone of growth at the interface between the agar and the polystyrene surface was measured. All motility experiments were performed in triplicate.

### Ability to form biofilm

Biofilm formation was assayed as described with modifications [19]. Briefly, *P. aeruginosa *strains were grown in 96-well plates in M63 supplemented with 0.5% casamino acids and 0.2% glucose. Plates were incubated at 37°C under mild shaking at 50 rpm (C24 Incubator Shaker, New Brunswick Scientific, Edison, NJ) for 8 hrs. The wells were then rinsed thoroughly with water and the attached material was stained with 0.1% crystal violet, washed with water, and solubilized in ethanol. Solubilized fractions were collected and absorbance measured at 550 nm with a Plate Reader. All experiments were performed in triplicate.

### Adhesiveness

Caco-2 cells were grown to confluence in 24-well plates using HEPES-buffered DMEM media containing 10% fetal bovine serum. Overnight cultures of *P. aeruginosa *were added to the apical side of Caco-2 cells to a final concentration of 10^7 ^CFU/ml and co-incubated for 1 hour at 37°C, 5% CO_2_. Following the one hour incubation, the media was removed and ten-fold dilutions were plated on PIA plates to quantify non-adherent bacteria. Wells were then washed with a continuous flow of 35 ml of PBS. A final single washing with 200 μl was diluted and plated on PIA to quantify the final amount of remaining non-adherent bacteria. Caco-2 cells were then trypsinized with 200 μl Trypsin-EDTA (Gibco), incubated for 20 min at 37°C, 5% CO_2_, and lysed with 400 μl of a lysis mixture (PBS, EDTA 10 mM, Triton X-100 0.25%) [20] added directly to the trypsinized Caco-2 cells. The cells were vigorously pipetted for one minute, and released bacteria were plated on PIA to quantify adherent cells. The proportion of bacterial cells adhering to Caco-2 cells was calculated as (adherent cells - cells in last washing)/non-adherent + adherent cells. All experiments were performed in triplicate.

### Effect of exposure of MDR *P. aeruginosa *clinical isolates to Gm on growth rate

Overnight culture of *P. aeruginosa *clinical isolate #1 was diluted as 1:100 in fresh M63 media supplemented with 0.5% casamino acids and 0.2% glucose and grown for 2 hours. After that, culture was spitted for control (no Gm) and Gm-variant that was added by Gm to a desirable concentration. 300 μl aliquots (in triplicates) were loaded in 96-well plate, and absorbance at OD550 nm was measured dynamically during growth at 37°C, 200 rpm. All experiments were performed in triplicate.

### The *exoU *and *exoS *gene detection by PCR

PCR assays for detection of the *exoU *and *exoS *genes were performed using intact *P. aeruginosa *grown on PIA as a source of template chromosomal DNA as described [16]. Amplification was performed in the presence of primers for *exoU*: *exoU*2998, 5'-GCTAAGGCTTGGCGGAATA-3' and *exoU*3182, 5'-AGATCACACCCAGCGGTAAC-3'; for *exoS*: *exoS *1106, 5'-ATGTCAGCGGGATATCGAAC-3', and *exoS *1335, 5'-CAGGCGTACATCCTGTTCCT-3'.

### Cytotoxicity assay

Caco-2 cells were grown to confluence in 96-well plates, and inoculated apically by *P. aeruginosa *to the final concentration of 10^7 ^CFU/ml. Cells were incubated at 37°C, 5% CO_2_, for 8 hours, and released lactate dehydrogenase was determined by CytoTox 96 assay (Promega). All experiments were performed in triplicate.

### Statistical analysis

Statistical analysis of the data was performed using Student t-test. Regression analysis was performed using Sigmaplot software.

## Results

### Morphological and demographic analyses of MDR *P. aeruginosa *clinical isolates

Morphological and demographic data are displayed in Table 1. *P. aeruginosa *strains were consecutively collected based on their resistance to gentamicin (Gm), however most clinical isolates displayed multiple antibiotic resistances to various antibiotics clinical used against *P. aeruginosa*. Most strains were obtained from sputum and tracheal aspirates while few were from tissues and urine. Significant variation was noted in colony morphology among the various strains. Environmental strains PA190 and PA180 were also tested for antibiotic resistance. Results indicated that PA190 was sensitive to all of the antibiotics routinely used for *P. aeruginosa *infection, whereas PA180 was resistant to Gm.

**Table 1:** Demographic and morphological data of MDR *P. aeruginosa *isolates

##	Morphology of colony on PIA	Antibiotic resistance ^a^	Source	Patient location
1	Yellow, smooth, flat edge	IMI 11, Ptaz14, Cefr 16, Ctaz 17, Gm 6, Tobr 6, Amik 18, Cipr 6 [b]	DN^c^	DN^c^
2	Green, smooth, flat edge	Tobr 16, Cipr 4, Gm 16, Ptaz 128 [d]	Sputum	ICU
4	Slightly green, rough edge	IMI 16, Ctaz 64, Gm 16, Ptaz 128 [d]	Tracheal aspirate	ICU
6	Bright greenish-blue, smooth, flat edge	Gm 16, Ptaz 128, Tobr 16 [d]	Tracheal aspirate	Burn ICU
7	Green, smooth, flat edge	Gm 16, Cipr 4, Tobr 16 [d]	Wound	Floor
8	Green, rough edge	IMI 21, Ptaz 28, Cefr 24, Ctaz 27, Gm 6, Tobr 9, Amik 6, Cipr 26 [b]	Maxillary sinus	ENT clinic
9	Greenish-blue, slightly roug,	Gm 16, Cipr 4, Tobr 16, Ptaz 128, Levo 8 [d]	Clean void urine	Floor
10	White, smooth, flat edge, mucoid	Gm 16, Cipr 4, Tobr 16 [d]	Sputum	Burn ICU
11	Slightly green, flat edge	Gm 10, Amik 13, Tobr 11, IMI 6, Ptaz 14 [b]	Sputum, CFRC	Pulmonary
12	Green, rough, nonflat edge	Gm 11 [b]	Sputum, CFRC	Floor
13	Bright yellow, smooth, flat edge	Gm 16, Cipr 4, Tobr 16 [d]	Catheter tip	Floor
14	Bright yellow, smooth, flat edge	Gm 16, Cipr 4, Tobr 16 [d]	Catheter tip	Floor
15	Green, slightly rough, nonflat edge	Gm 16, Cipr 4, Tobr 16, Ptaz 128, Levo 8, Amik 64, Ctaz 64, IMI 16 [d]	Urine	Nursing home
16	Green, slightly rough, nonflat edge	Gm 6, Cipr 6, Tobr 10, Ptaz 17, Amik 6, Ctaz 10 [b]	Sputum, CFRC	Pulmonary
17	White, mucoid	Gm 8, Cipr 15, Ptaz 15, Amik 8 [b]	Sputum, CFRC	Pulmonary
18	Yellow, smooth, flat edge	Gm 16, Tobr 16 [d]	Catheter tip	Burn ICU
19	Slightly green, smooth, flat edge	IMI 23, Ptaz 35, Cefr 24, Ctaz 30, Gm 9, Tobr 15, Amik 11 [b]	ET tube	Burn ICU
20	Slightly green, smooth, flat edge	IMI 8, Ptaz 21, Cefr 20, Ctaz 22, Gm 12, Tobr 17, Amik 17 [b]	Tracheal aspirate	ICU
21	Slightly green, smooth, flat edge	Gm 16, IMI 16 [d]	Tracheal aspirate	ICU
22	Green, smooth, flat edge	Gm16 [d]	Wound	Floor
23	Slightly green, smooth, flat edge	Gm16 [d]	Tracheal aspirate	Burn ICU
25	Rough, nonflat edge, slightly green	Ctaz 64, IMI 16, Gm 16 [d]	Tracheal aspirate	ICU
26	Rough, nonflat edge, slightly green	Ctaz 64, IMI 16, Gm 16 [d]	Tracheal aspirate	ICU
27	Rough, nonflat edge, slightly green	Ctaz 64, IMI 16, Gm 16, Ptaz 128 [d]	Urine	ICU
28	Rough, nonflat edge, slightly green	Ctaz 64, IMI 16, Gm 16 [d]	Foley catheter urine	ICU
29	Green, slightly rough, nonflat edge	Amik 6, Tobr 12, Gm 6 [b]	Sputum, CFRC	Pulmonary
30	Pink, smooth, flat edge, mucoid	IMI 29, Ptaz 22, Cefr 6, Ctaz 24, Gm 6, Tobr 6, Amik 6, Cipr 19 [b]	Sputum, CFRC	Pulmonary
31	Pink, smooth, flat edge, mucoid	IMI 27, Ptaz 26, Cefr 19, Ctaz 27, Gm 6, Tobr 6, Amik 6, Cipr 26 [b]	Sputum, CFRC	Pulmonary
33	Slightly green, smooth, flat edge	Amik 13, IMI 6, Gm 9, Cipr 6 [b]	Clean void urine	Floor
34	Slightly green, smooth, flat edge	Gm 16, Cipr 4, Tobr 16, Ptaz 128, Ctaz 64, IMI 16 [d]	Tissue	Floor
35	Rough, nonflat edge, slightly green	Gm 16, Cipr 4, Tobr 16, Ptaz 128, Ctaz 64, IMI 16 [d]	Tissue	Floor

^a ^Cephems: ceftazidime (Ctaz), cefoperazone (Cefr); carbapenems: imipenem (IMI); aminoglycosides: amikacin (Amik), tobramycin (Tobr), gentamicin (Gm); fluoroquinolones: ciprofloxacin (Cipr); and b-lactam/b-lactamase inhibitor combinations: piperacillin/tazobactam (Ptaz); [b], performed by disc diffusion method; ^c ^DN: demographic data are not available; [d], performed by MIC on Vitek 2.

### RAPD fingerprinting of consecutively obtained MDR *P. aeruginosa *clinical isolates

**Figure 1:**
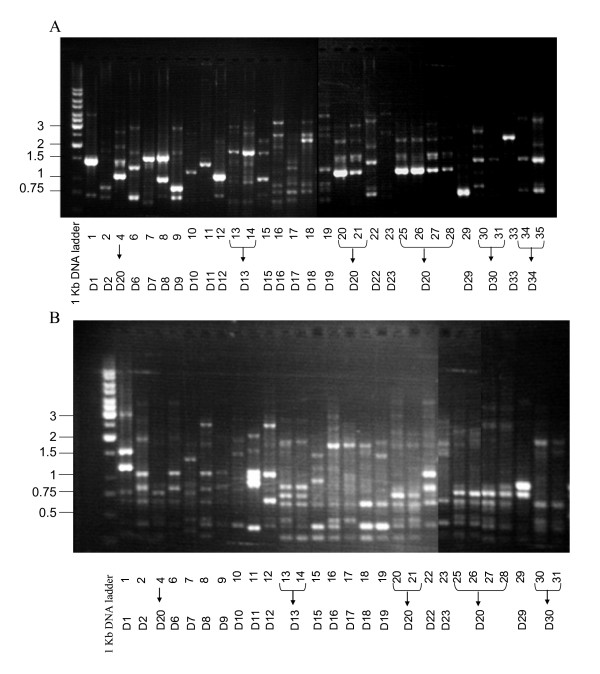
**Random Amplified Polymorphic DNA Typing (RAPD) of multi-drug resistant (MDR) *P. aeruginosa *clinical isolates**. Random Amplified Polymorphic DNA Typings were generated by RAPD primers (A) 208, 5'ACGGCCGACC 3', and (B) 272, 5'AGCGGGCCAA3' [15]. Molecular size markers (Fermentas) were run in left lanes, and DNA sizes (in kilobases) are indicated to the left of the gels.

A total of 31 *P. aeruginosa *clinical isolates were typed by RAPD analysis with primers 208 (Fig. 1A) and 272 (Fig. 1B) [15]. RAPD fingerprints demonstrated that most clinical strains were of distinct RAPD type. More detailed demographic analysis of strains with similar RAPD revealed that strains 13 and 14 (G13) were from a single patient, strains 30 and 31 (G30) were also from a single patient, and 34 and 35 (G34) were also from a single patient. RAPD fingerprint G20 was similar for strains 4, 20, 21, and 25–28. All of these strains were obtained from specimens of tracheal aspirate, urine, and Foley catheter urine from the same patient during a 4 month period. As such, the total 31 clinical isolates contained 22 different genotypes.

### Effect of multi-drug resistant (MDR) clinical isolates of *P. aeruginosa *on transepithelial resistance (TER) of Caco-2 monolayers

Among clinical isolates in our study, three isolates, #12, #22, and #23 showed resistance to Gm only, and two isolates, #18 and #21 showed resistance to only two anti-pseudomonas antibiotics (Table 1). Since multi-drug resistance is generally defined as resistance to three or more antimicrobial agents [10], we did not include these strains in any further experiments. Strains 13 and 14, 30 and 31, 34 and 35 were found to be repeat isolates based on RAPD analysis and demographic data; therefore, strains 14, 31, and 34 were not included in any further experiments.

**Figure 2:**
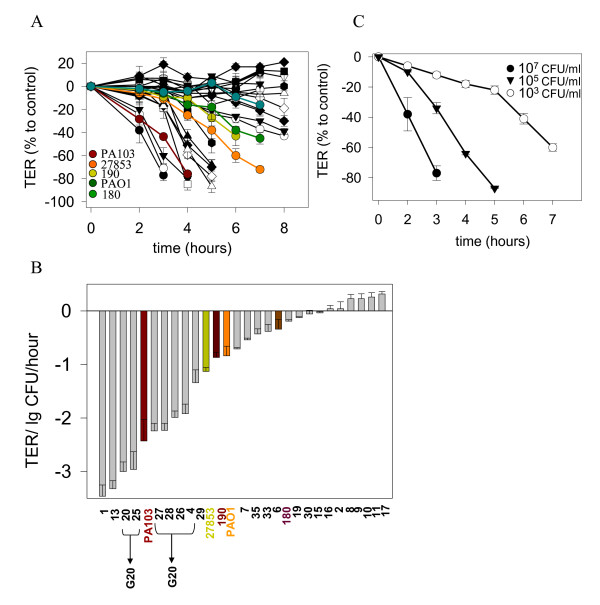
**Effect of multi-drug resistant (MDR) clinical isolates of *P. aeruginosa *on transepithelial resistance (TER) of Caco-2 monolayers**. (A) TER of Caco-2 cells measured dynamically during co-incubation with MDR *P. aeruginosa*. PA103, well known cytotoxic strain; PAO1, well known invasive laboratory strain; ATCC 27853, a prototype laboratory strain used as a susceptible control in the antibiotic resistance assay; 190, a Gm^S ^environmental isolate; and 180, a Gm^R ^environmental isolate were used as non-MDR controls. TER is expressed as % of control TER in confluent Caco-2 cells. (B) MDR clinical isolates and control non-MDR *P. aeruginosa *strains are arranged in descending order of their ability to affect the TER of Caco-2 cells expressed as ΔTER/hour normalized to the initial bacterial cell density. (C) The most virulent strain, #1, induced a fall in TER even at an extremely low concentration of 10^3 ^CFU/ml. Data are mean ± SD (n = 3).

The effect of MDR *P. aeruginosa *clinical isolates on TER of Caco-2 cells following apical inoculation is summarized in Figure 2. Dynamic tracking of TER following apical exposure of Caco-2 cells to *P. aeruginosa *(Fig. 2A) (Fig. 2B) demonstrated that the strains 1, 13, and that of RAPD type G20 induced a rapid and profound decrease in TER similar to the highly cytotoxic strain PA103 [21]. Three isolates, 29, 7, and 15 had significant yet moderate effect on TER similar to the antibiotic sensitive reference strains ATCC 27853, PA01, and PA190. The remaining strains showed a minimal to negligible effect on TER as did the Gm^R ^environmental isolate, PA180. Strain #1 was found to be most virulent strain based on the TER response of Caco-2 cells. TER decreased following apical exposure to as little as 10^3 ^CFU/ml (Fig. 2C) suggesting a profound ability of the organism to disrupt epithelial barrier function.

### Adherence properties, motility patterns, and biofilm formation in relation to the epithelial barrier-disrupting phenotype

**Figure 3:**
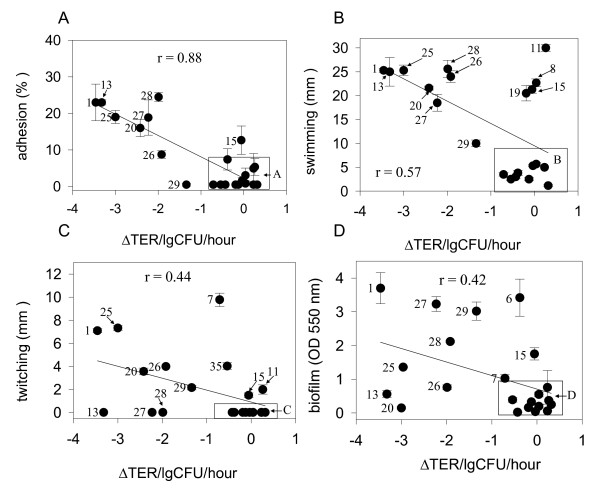
**Correlation of the ability of MDR *P. aeruginosa *clinical isolates to induce decrease in TER with phenotypic features**. (A) adhesion, (B) swimming motility, (C) twitching motility, and (D) biofilm formation. Strains with numerically close values are grouped into enclosed boxes. Data are mean ± SD (n = 3).

Regression analysis revealed that adherence (Fig. 3A) and swimming motility (Fig. 3B) significantly correlated with the TER changes in Caco-2 cells induced by MDR *P. aeruginosa *(r = 0.88, P < 0.0001, r = 0.57, P < 0.01, respectively). There was no correlation however between TER changes and twitching motility (r = 0.44) (Fig. 3C), or biofilm formation (r = 0.42) (Fig. 3D). High swimming motility and adherence to Caco-2 cells were the main phenotypic features of MDR barrier-disruptive strains 1, 13, and strains of G20 RAPD fingerprint. As a group, strains with a minimal effect on TER were characterized as having attenuated adherence, motility, and biofilm formation although several strains with a minimal effect on TER did display high motility behavior suggesting that motility alone is not predictive of the virulence of MDR *P. aeruginosa *against the intestinal epithelium.

### Effect of exposure of MDR *P. aeruginosa *to Gm on growth rate

**Figure 4:**
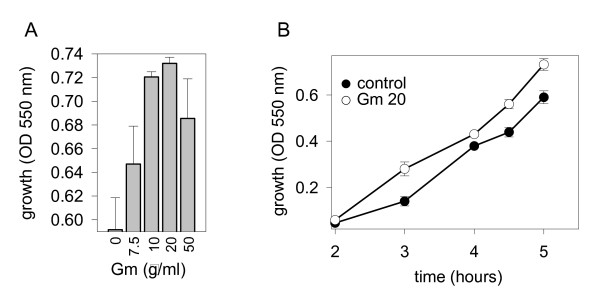
**Effect of exposure to Gm on the growth of *P. aeruginosa *clinical isolate #1**. (A) Cell density measured as absorbance at 550 nm after 5 hours of growth in the presence of varied concentration of Gm. (B) Dynamically tracked cell density of clinical isolate #1 grown in the absence (control) or presence of Gm, 20 μg.ml^-1^. Data are mean ± SD (n = 3).

Strains #1, 13, and those of G20 RAPD genotype, the most virulent in terms of their effect on TER were tested for their ability to grow in the presence of Gm. We found that as much as 50 μg.ml-1of Gm had no effect on the growth of strains 13 and G20 RAPD genotype strains (data not shown), whereas strain #1 grown in the presence of Gm showed a dose-dependent stimulation (10–20 μg.ml^-1^) of growth (Fig. 4A). Dynamic tracking of strain #1 exposed to 20 μg.ml^-1 ^of Gm demonstrated this effect to be greatest during the exponential phase of growth (Fig. 4B).

### Cytotoxicity of MDR *P. aeruginosa *clinical isolates, correlation with *exoU/exoS *genotype

**Figure 5:**
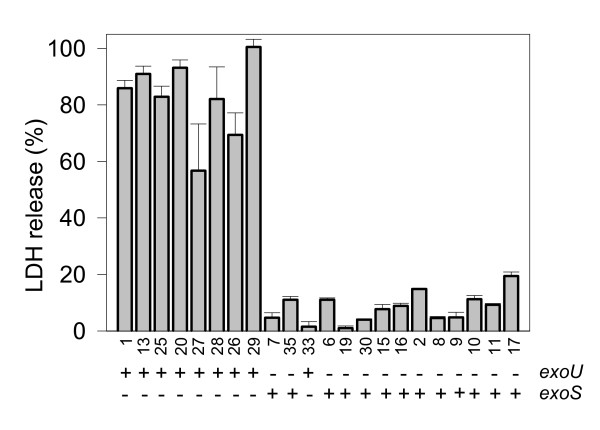
**Cytotoxicity of MDR *P. aeruginosa *clinical isolates against Caco-2 monolayers and their correlation to the *exoU/exoS *genotype**. Cytotoxic effect on Caco-2 monolayers was determined after 8 hours of co-incubation and correlated to the *exoU*-containing clinical isolates with the exception of isolate #33. Data are mean ± SD (n = 3).

The cytotoxic effect of the various clinical isolates following 8 hours of bacterial exposure is shown in Figure 5. Results demonstrated that most MDR clinical isolates with barrier-disruptive phenotypes harbored the *exoU *gene (except strain #33) and displayed cytotoxicity against Caco-2 monolayers. Clinical isolates harboring the *exoS *gene were not cytotoxic to Caco-2 cells.

## Discussion

### Effect of MDR *P. aeruginosa *clinical isolates on the intestinal epithelial barrier

Numerous reports have documented that the rise in multi-drug resistant nosocomial pathogens continues to threaten hospitalized patients despite various countermeasures including isolation techniques and antibiotic de-escalation therapy [22,23]. In the present study we focused on the effect of multi-drug resistant strains of *P. aeruginosa *on the intestinal epithelial barrier since intestinal *P. aeruginosa *has been shown to be a major cause of morbidity and mortality among immuno-compromised patients [4,24,25].

Caco-2 cells are an ideal cell model for these studies since they express several markers that are characteristic of normal intestinal epithelial cells including the presence of a brush border and the ability to maintain a highly resistant barrier to bacterial pathogens [17,26]. As previously mentioned, the ability of microorganisms to adhere to and alter the barrier function of intestinal epithelia is a key feature that defines their pathogenicity within the intestinal tract reservoir [27,28]. Conversely, the ability of the epithelium to resist the barrier dysregulating effect of a given pathogen through the release of mucus, IgA, defensins, etc, defines its innate defensive properties [29-31]. During host illness, especially under circumstances of critical illness, this delicate balance can be tipped in the favor of the microbe where the potential for a versatile pathogen like *P. aeruginosa *to subvert and erode an already compromised epithelial defense system exists [8,32].

Whether MDR *P. aeruginosa *[33-36] strains necessarily express a more virulent phenotype continues to remain a controversial issue. While the behavior of MDR *P. aeruginosa *against the intestinal epithelium is unknown, its high prevalence in the intestinal tract of critically ill and immuno-compromised patients begs a better understanding of the degree to which certain strains can disrupt the intestinal epithelial barrier. For example the apical side of the intestinal epithelium is highly resistant to various toxic and cytolytic exoproducts of *P. aeruginosa *including exotoxin A and elastase [8,11,37], whereas the lung is highly susceptible. As such, lung models of *P. aeruginosa *infection and pathogenesis cannot be directly extrapolated to the intestinal model. Interestingly, data from the present study establish that among the MDR *P. aeruginosa *isolates tested in the Caco-2 model, most display a minor to minimal ability to disrupt the intestinal epithelium in both motile and non-motile strains.

### Phenotype and genotype analysis of *P. aeruginosa *isolates highly disruptive to the intestinal epithelium

We identified 8 MDR clinical isolates with 3 distinct RAPD fingerprints that display a disruptive phenotype against the intestinal epithelial barrier. The presence of such strains within the intestinal tract of critically ill patients has the potential to induce a state of gut-derived sepsis with a high mortality rate as their presence in this site is often difficult to detect and eradicate.

Common features of these highly disruptive strains include high swimming motility, increased adhesiveness to intestinal epithelium, and the presence of the *exoU *gene. ExoU, an effector protein of the type III secretion machinery, has been previously shown to play a major role in mediating a cytotoxic phenotype of *P. aeruginosa *[38,39] against lung epithelial cells and HeLa cells [40]. That ExoU also plays an important role in disruption of the intestinal epithelial barrier and cellular cytotoxicity in this model suggests that intestinal colonization with MDR *P. aeruginosa *strains harboring the *exoU*-genotype may be associated with poor outcome in patients colonized by such strains. Although the presence of ExoS has been previously reported to play a role in the virulence of *P. aeruginosa *in a lung model [41], we found no correlation between exoS-genotype and the ability of strains to disrupt the intestinal epithelial barrier among our clinical isolates. As previously reported and confirmed by the results of the present study [42], motility and adhesion to host cells are important factors that appear to predict virulence.

As we and others have suggested, bacteria are fully capable of changing their virulence phenotype in direct response to host illness [43,44]. The frequent use of multiple antibiotics in the most severely ill patients could lead to the acquisition of, or alternatively the transformation to, highly virulent strains of *P. aeruginosa *that pose a significant threat to the patient. The ability of multi-drug resistant strains to persist for prolonged periods in such patients may allow for the development of extremely virulent phenotypes [45].

In conclusion, heterogeneity among critically ill humans, variability in immune response, and antibiotic use could explain the extremely polar phenotypes identified in the series of multi-drug resistant isolates collected in the present study: from phenotypes that are essentially inert with respect to the intestinal epithelium to highly motile, adhesive, and destructive phenotypes. Phenotypic assays such as motility and adhesiveness, and genotyping for the *exoU *gene could provide a significant prognostic input to identify multi-drug resistant *P. aeruginosa *strains most likely to place patients at risk for lethal gut-derived sepsis. Further characterization of strains 1, 13 and those of G20 RAPD genotype will be necessary to better understand the precise mechanism by which these strains disrupt the intestinal epithelium to a degree not previously reported for any intestinal pathogen.

## Abbreviations

Multi-drug resistance, MDR; transepithelial resistance, TER; random amplified polymorphic DNA PCR fingerprinting, RAPD; phosphate buffered saline, PBS; lactate dehydrogenase, LDH; *Pseudomonas *isolation agar, PIA.

## Competing interests

The author(s) declare that they have no competing interests.

## Authors' contributions

OZ performed experimental design, most experimental work, and drafting/revising the manuscript. JEK had developed and carried out the adhesiveness assay. YW was responsible for cultivation of Caco-2 cells and growing them on transwells. CB isolated and identified clinical isolates. OS participated in adherence and RAPD analyses. LW participated in adherence analyses. JRT was involved in the experimental design and discussion of experiments and manuscript revision. JCA performed experimental design, experimental data discussion, drafting/revising the manuscript, and is the PI of the NIH funding mechanism of the study. All authors read and approved the final manuscript.
